# In Vivo and In Silico Approach on Valproic Acid-Induced Cardiac Damage and Protection by *Moringa oleifera* Lam

**DOI:** 10.3390/ph19101561

**Published:** 2026-10-01

**Authors:** Elmas Irmak Cil Arslan, Ismet Burcu Turkyilmaz Mutlu, Umar Faruk Magaji, Refiye Yanardag, Ozlem Sacan

**Affiliations:** 1 Institute of Graduate Studies, Istanbul University-Cerrahpaşa, 34320 Avcilar, Istanbul, Türkiye; irmakcil@gmail.com; 2 Department of Chemistry, Faculty of Engineering, Istanbul University-Cerrahpaşa, 34320 Avcilar, Istanbul, Türkiye; yanardag@iuc.edu.tr; 3 Department of Biochemistry and Molecular Biology, Federal University Birnin Kebbi, Birnin Kebbi 860101, Kebbi State, Nigeria; umar.faruk@fubk.edu.ng

**Keywords:** *Moringa oleifera*, valproic acid, heart, antioxidants, rat

## Abstract

**Background/Objectives**: Valproic acid (sodium salt form, SVA) is a short-chain fatty acid used to treat epilepsy. Nevertheless, its side effects reach a toxic level during treatments. *Moringa oleifera* Lam. (Moringa) is a traditional plant that provides protection against various diseases due to its antioxidant components. In this study, the protective and ameliorative effect of Moringa on SVA-induced heart damage via in vivo and in silico approach was investigated. **Methods**: Experimental animals were split into four groups: Control, Moringa extract given group [300 mg.kg^−1^], SVA given group [500 mg.kg^−1^] and SVA + Moringa given group [at the same doses and time]. The procedure was carried out for fifteen days, after an overnight fasting, all animals were sacrificed under anesthesia. The heart tissues were taken, homogenized with cold physiological saline, and then centrifuged. **Results**: In clear supernatants, reduced glutathione, advanced oxidized protein products, total oxidant status, reactive oxygen species, oxidative stress index, nitric oxide, glycoproteins levels and glutathione-dependent enzymes, gamma glutamyl transferase, glutamate oxalacetate transaminase, myeloperoxidase and carbonic anhydrase were increased. Conversely, total antioxidant capacity levels, catalase, superoxide dismutase, xanthine oxidase, lactate dehydrogenase, sodium potassium ATPase and alkaline phosphatase activities were decreased after SVA exposure. Treatment with Moringa significantly suppressed SVA-induced heart injury; oxidative stress parameters, total antioxidant capacity levels, the activity of antioxidant defense enzymes, and the activity of the enzymes critical for the cardiac metabolism were improved, while pH and cellular ion balance were regulated. **Conclusions**: Moringa extract-prevented anomalies and ameliorated biochemical alterations by decreasing oxidative stress.

## 1. Introduction

Valproic acid [VPA] is one of the most preferred drugs for epilepsy treatments and various neuropsychiatric diseases. Despite its effectiveness, the use of VPA is limited due to its well-known side effects. VPA associated oxidative stress have been investigated for a long time. The main target of VPA induced oxidative stress tends to be the liver and other organs like kidney, stomach, etc., [1,2,3]. The alteration of redox balance in favor of oxidants (like lipid peroxidation products and carbonyl derivatives, etc.) [4] is also inevitable during long-term usage of VPA.

The relationship between heart and VPA has been controversial. Some researchers have reported that VPA has protective effect on heart tissue under several circumstances. These are mostly related the histone deacetylase inhibitory effect of VPA, its oxidative stress reducing effect, cardiomyocyte hypertrophy diminishment, etc. [5]. However, other reports indicates that both VPA and its sodium salt form, sodium valproate [SVA], cause damage to heart tissue via inhibition of beta oxidation, as well as oxidative stress arising from the liver when exposed to the drug [6]. In general, energy metabolism is much more important for heart and the protection of mitochondrial energy flux is in question.

*Moringa oleifera* Lam. (Moringa) is consumed as food and used as folk medicine for a very long time. Every part of the plant (i.e., its leaves, roots, seeds, flowers etc.) has unique phytochemicals that include flavonoids, phenolic compounds, carotenoids, vitamins, sulfur containing amino acids, fatty acids [7,8]. The beneficial effects of Moringa are attributed to its antioxidant and redox decreasing effect on different tissues, antilipase activity, antiacetylcholine esterase effect [9,10,11]. Based on this information, Moringa extract was evaluated for its ability to reverse the deleterious effects of SVA on the heart tissue of rats.

## 2. Results

### 2.1. GC Results

Gas chromatography (GC) analysis was performed on Moringa leaves extract. According to the results, saturated fatty acids (C14:0, C16:0, C17:0, C18:0, C20:0, C22:0, C24:0), monounsaturated fatty acids (C16:1, C17:1, C20:1, C22:1, C24:1), and polyunsaturated fatty acids (C18:2, C18:3 and C20:3) were observed to be present.

### 2.2. Biochemical Results

**Figure 1:**
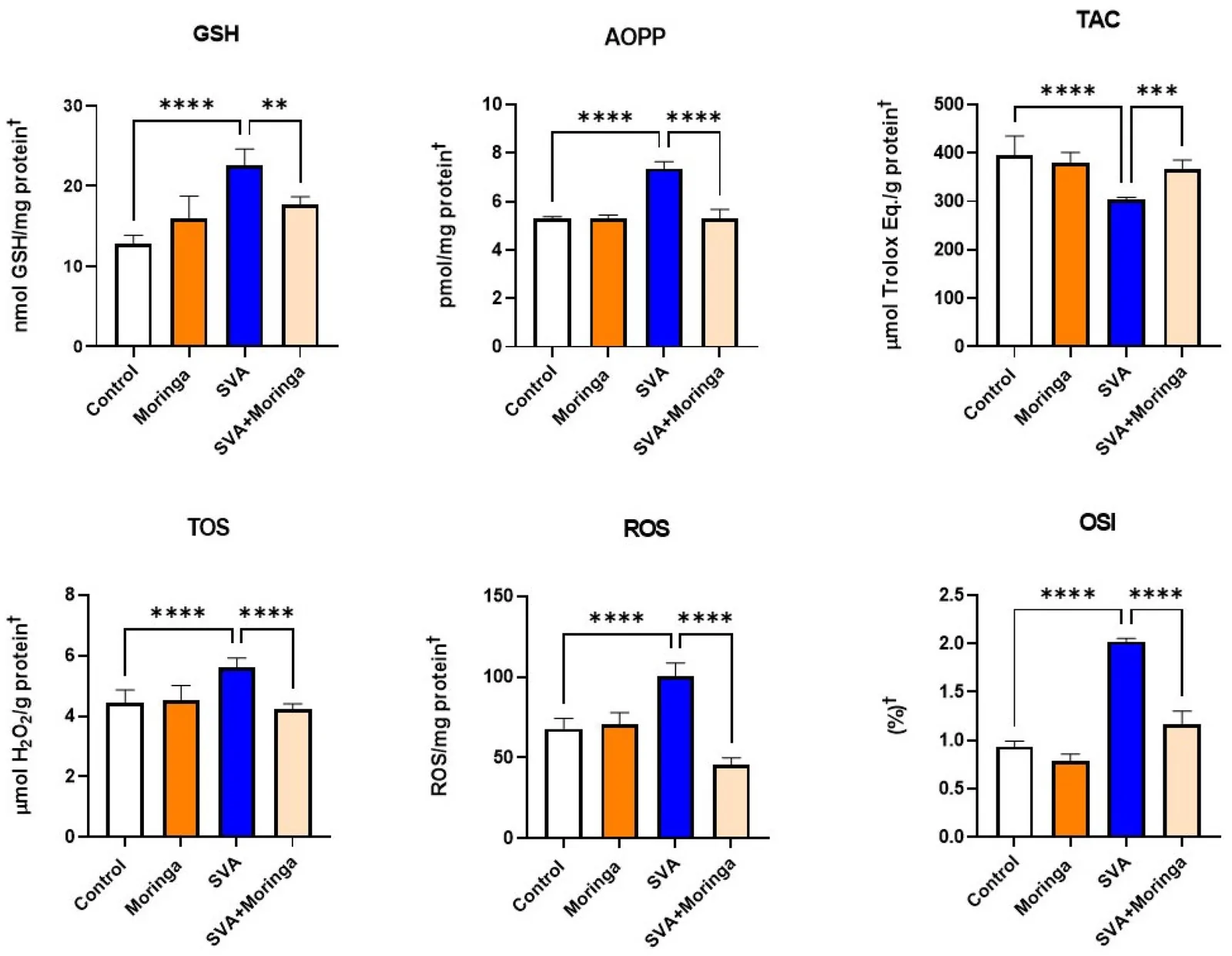
The heart GSH, AOPP, TAC, TOS, ROS and OSI levels of all experimental groups. ** *p* < 0.01, *** *p* < 0.001, **** *p* < 0.0001. ^†^ Values were expressed as the mean ± standard deviation.

The reduced glutathione (GSH), advanced oxidized protein products (AOPP), total antioxidant and total oxidant status (TAC and TOS, respectively), reactive oxygen species (ROS) and oxidative stress index (OSI) levels of all groups are presented in Figure 1. According to the results, SVA administration caused an increase in GSH, AOPP, TOS, ROS and OSI levels, while TAC levels were decreased in a significant manner compared to the control group (*p* < 0.0001). Moringa extract significantly reversed the levels of these parameters in the SVA + Moringa group as compared to the SVA group (*p* < 0.01, *p* < 0.001, *p* < 0.0001) (Figure 1).

**Figure 2:**
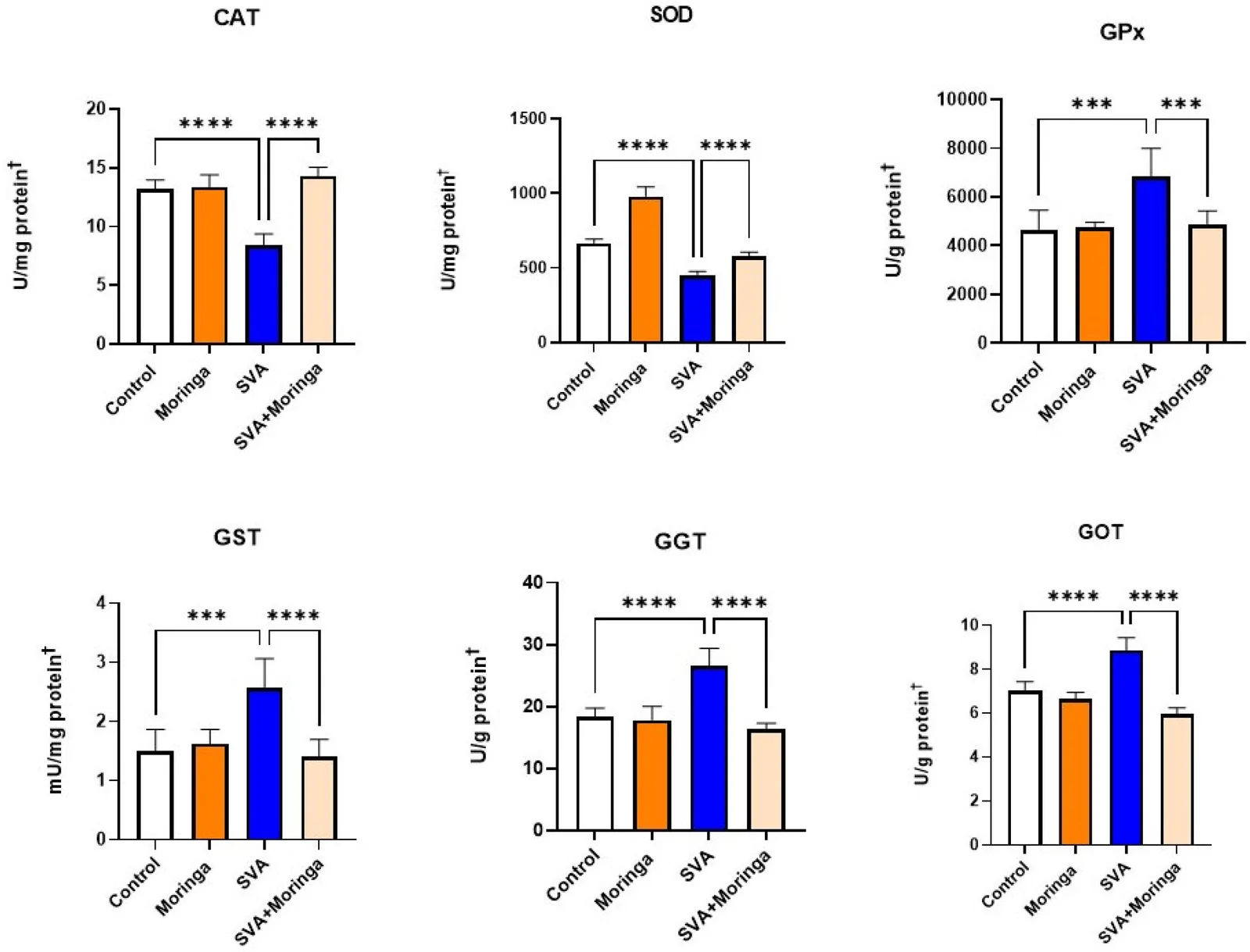
The CAT, SOD, GPx, GST, GGT and GOT activities of all experimental groups. *** *p* < 0.001, **** *p* < 0.0001. ^†^ Values were expressed as the mean ± standard deviation.

Catalase (CAT), superoxide dismutase (SOD), glutathione peroxidase (GPx), glutathione-S-transferase (GST), gamma glutamyl transferase (GGT) and, glutamate oxalacetate transaminase (GOT) activities of all groups are given in Figure 2. CAT and SOD activities decreased, while GPx, GST, GGT and, GOT activities were increased significantly following SVA administration as compared to the control group (*p* < 0.001, *p* < 0.0001). In the SVA + Moringa group, the activities of these enzymes were significantly reversed when compared to the SVA group (*p* < 0.001, *p* < 0.0001) (Figure 2).

**Figure 3:**
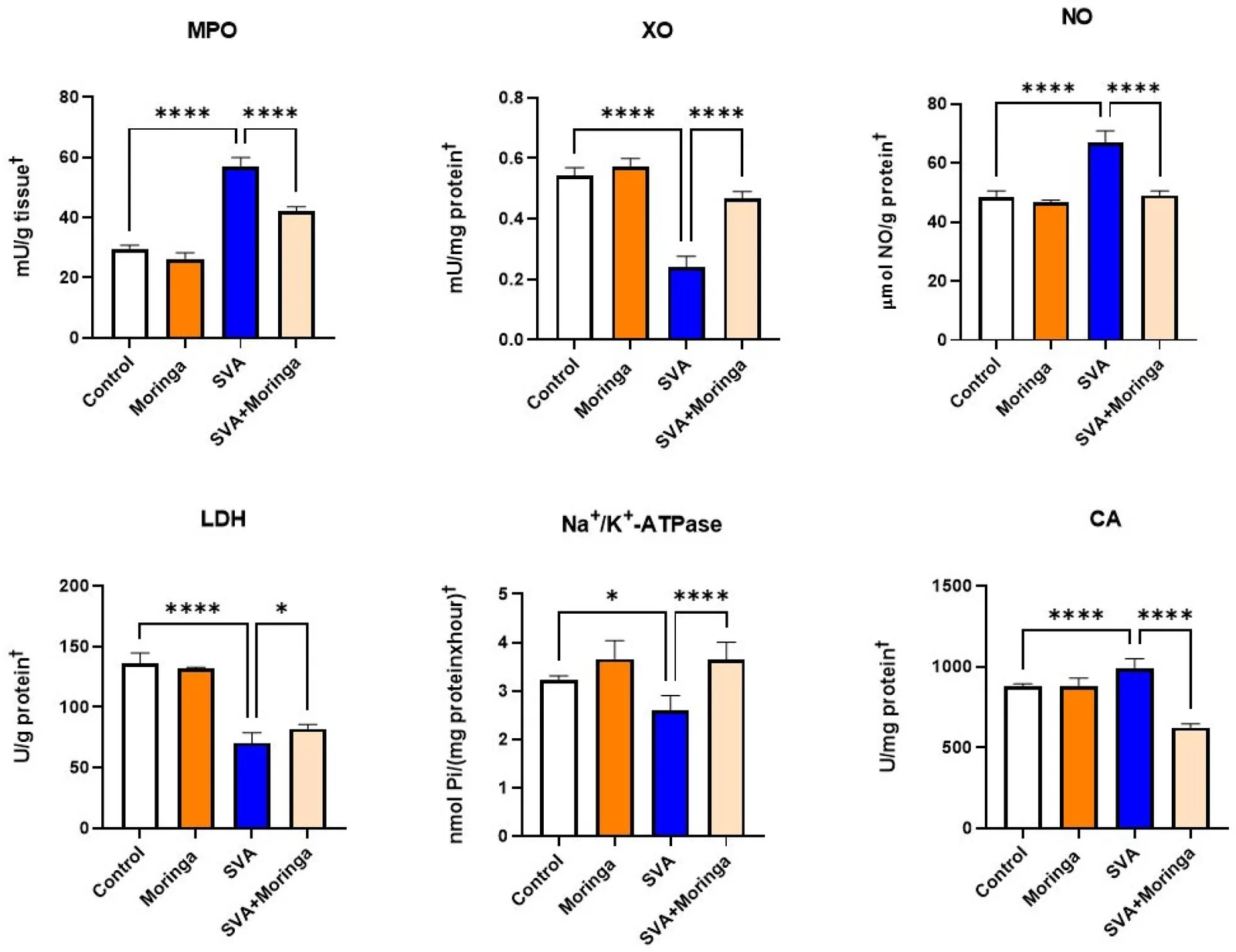
The MPO, XO, LDH, Na^+^/K^+^-ATPase, CA activities and NO levels of all experimental groups. * *p* < 0.05, **** *p* < 0.0001. ^†^ Values were expressed as the mean ± standard deviation.

Figure 3 presents myeloperoxidase (MPO), xanthine oxidase (XO), lactate dehydrogenase (LDH), sodium potassium ATPase (Na^+^/K^+^-ATPase), carbonic anhydrase (CA) activities, and nitric oxide (NO) levels of all groups. It was observed that the administration of SVA increased MPO and CA activities as well as NO levels. However, XO, LDH, and Na^+^/K^+^-ATPase activities were decreased in the control group (*p* < 0.05, *p* < 0.0001). When Moringa extract was given to the SVA group, their levels were significant reversed in the SVA + Moringa group as compared to the SVA group (*p* < 0.05, *p* < 0.0001) (Figure 3).

**Figure 4:**
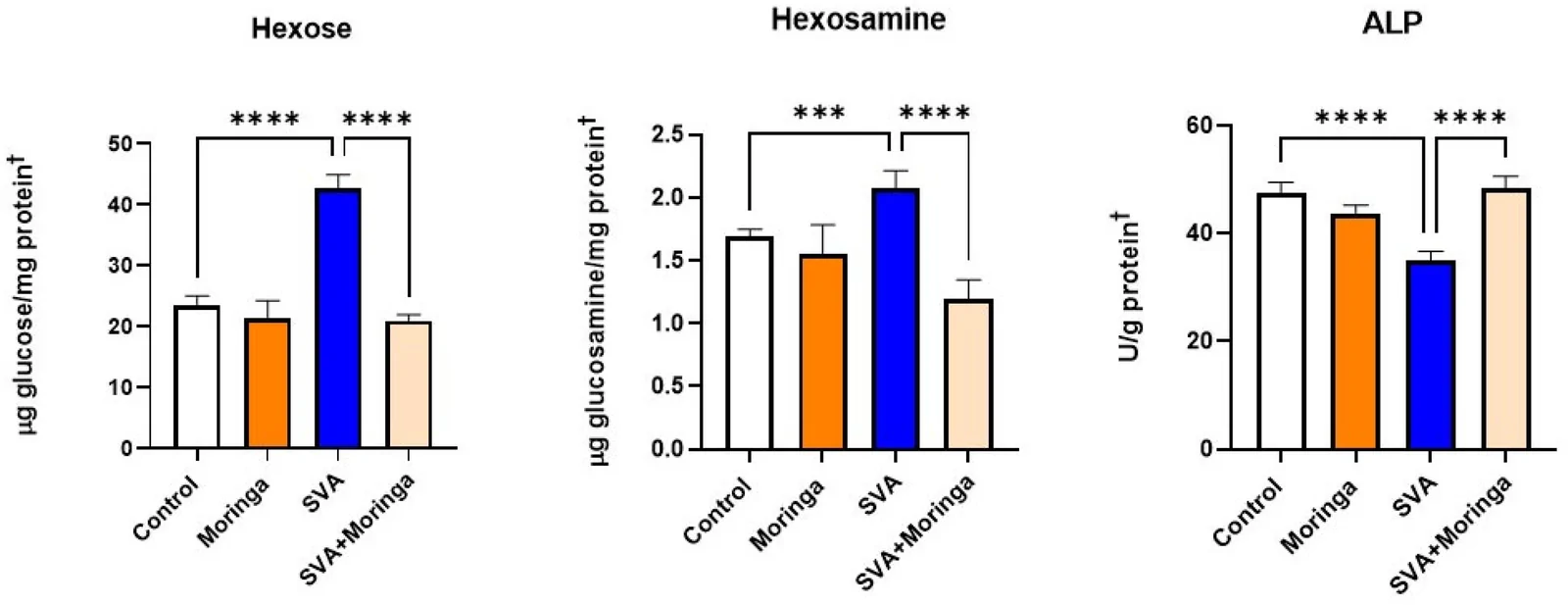
The hexose, hexosamine levels and ALP activities of all experimental groups. *** *p* < 0.001, **** *p* < 0.0001. ^†^ Values were expressed as the mean ± standard deviation.

As seen in Figure 4, SVA directly increased hexose and hexosamine levels, but decreased ALP activity compared with the control group (*p* < 0.001, *p* < 0.0001). The administration of Moringa extract significantly reversed effects in the SVA + Moringa group when compared with the SVA group (*p* < 0.001, *p* < 0.0001).

**Figure 5:**
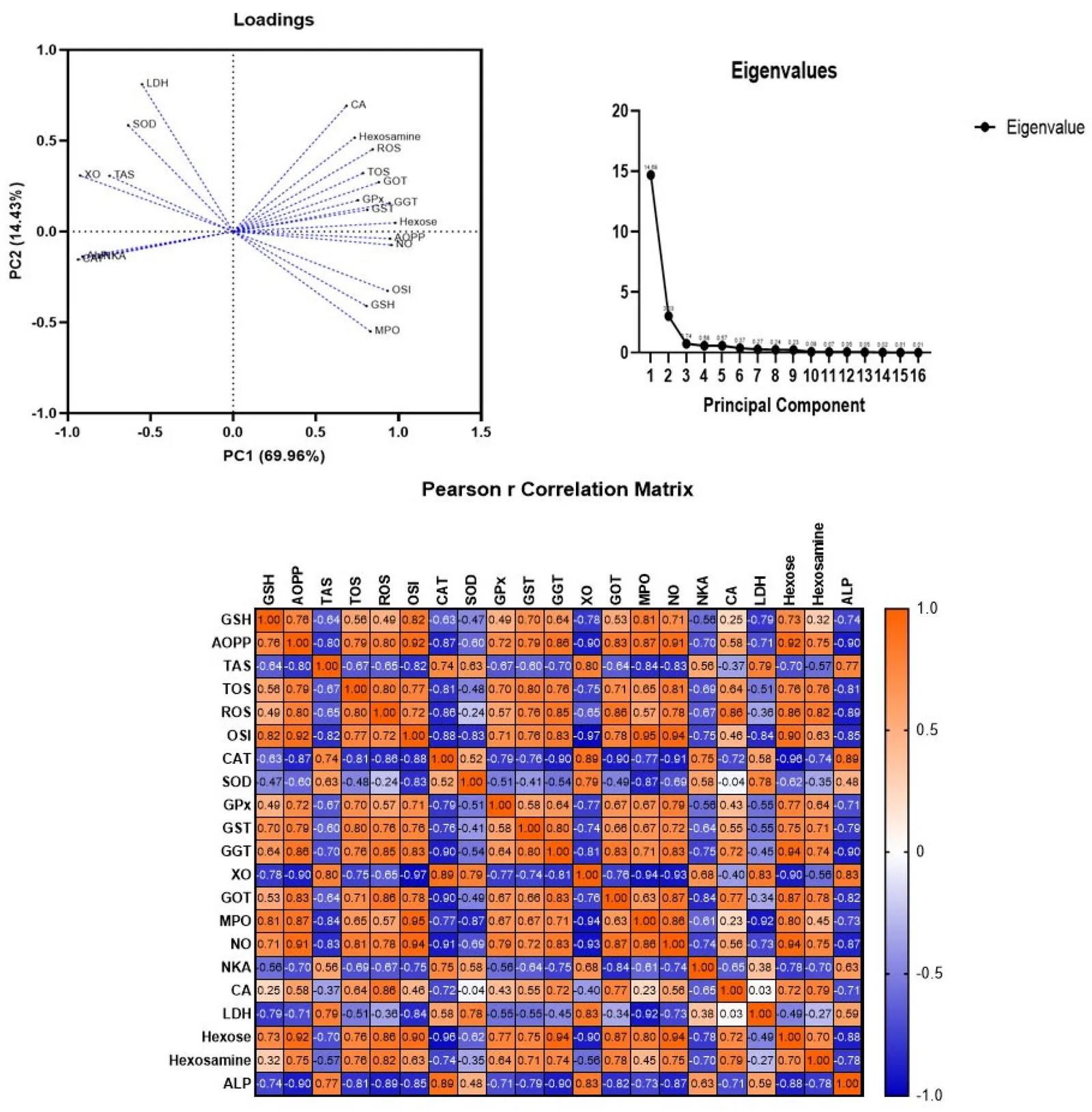
The PCA results of all biochemical parameters.

The principal component analysis (PCA) loadings, eigenvalue results, and correlation matrix chart are given in Figure 5. According to the results, the PC1, PC2, and total PCA values were 69.96%, 14.43%, and 84.39% respectively. LDH, SOD, XO, TAC, ALP, CAT and Na^+^/K^+^-ATPase were strongly in negative correlation with CA, glycoproteins, ROS, TOS, GOT, GPx, GGT, GST, AOPP, NO, OSI, GSH and MPO. In a Pearson r correlation matrix, a +1 value indicates the strongest positive correlation, while a −1 value indicates the strongest negative correlation. 0 value means there is no statistical correlation between any of two parameters. The current outcome (shown in the graphics of Figure 5) are consistent with the matrix results. Glycoprotein elevation and oxidative stress markers like AOPP showed high positive correlation. Additionally, the highest positive correlation was observed between oxidative stress marker enzymes and levels of non-enzymatic oxidative stress biomarkers. The best negative correlation (−0.97) was observed between XO and OSI (Figure 5).

### 2.3. In Silico Results

**Figure 6:**
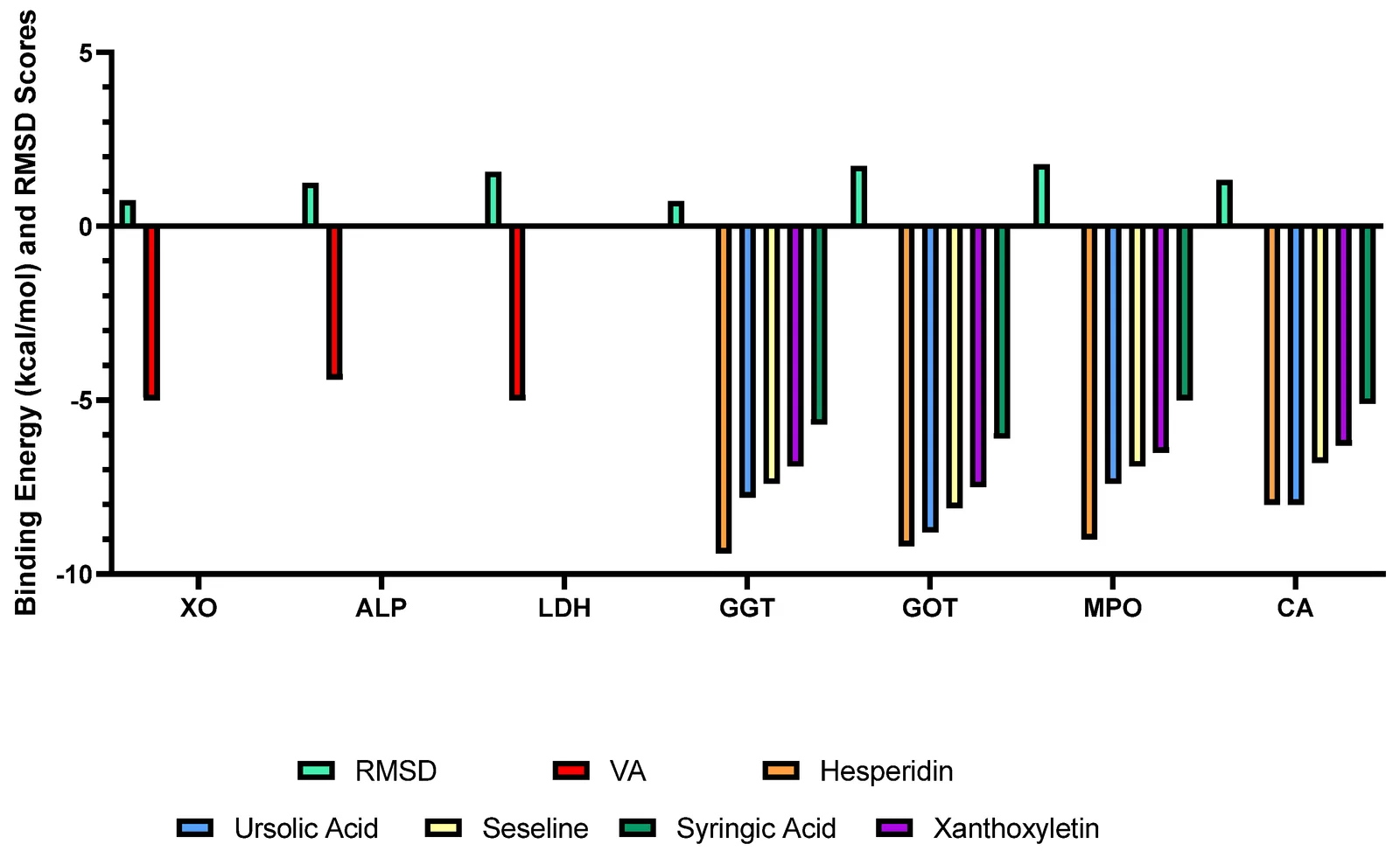
The binding energy values and RMSD scores.

The RMSD values and binding energy scores were given in Figure 6. The information for molecular docking studies is summarized in Table 1. XO and LDH showed the best binding score for VA. Among the natural compound found in Moringa, hesperidin exhibited the best binding capability.

**Figure 7:**
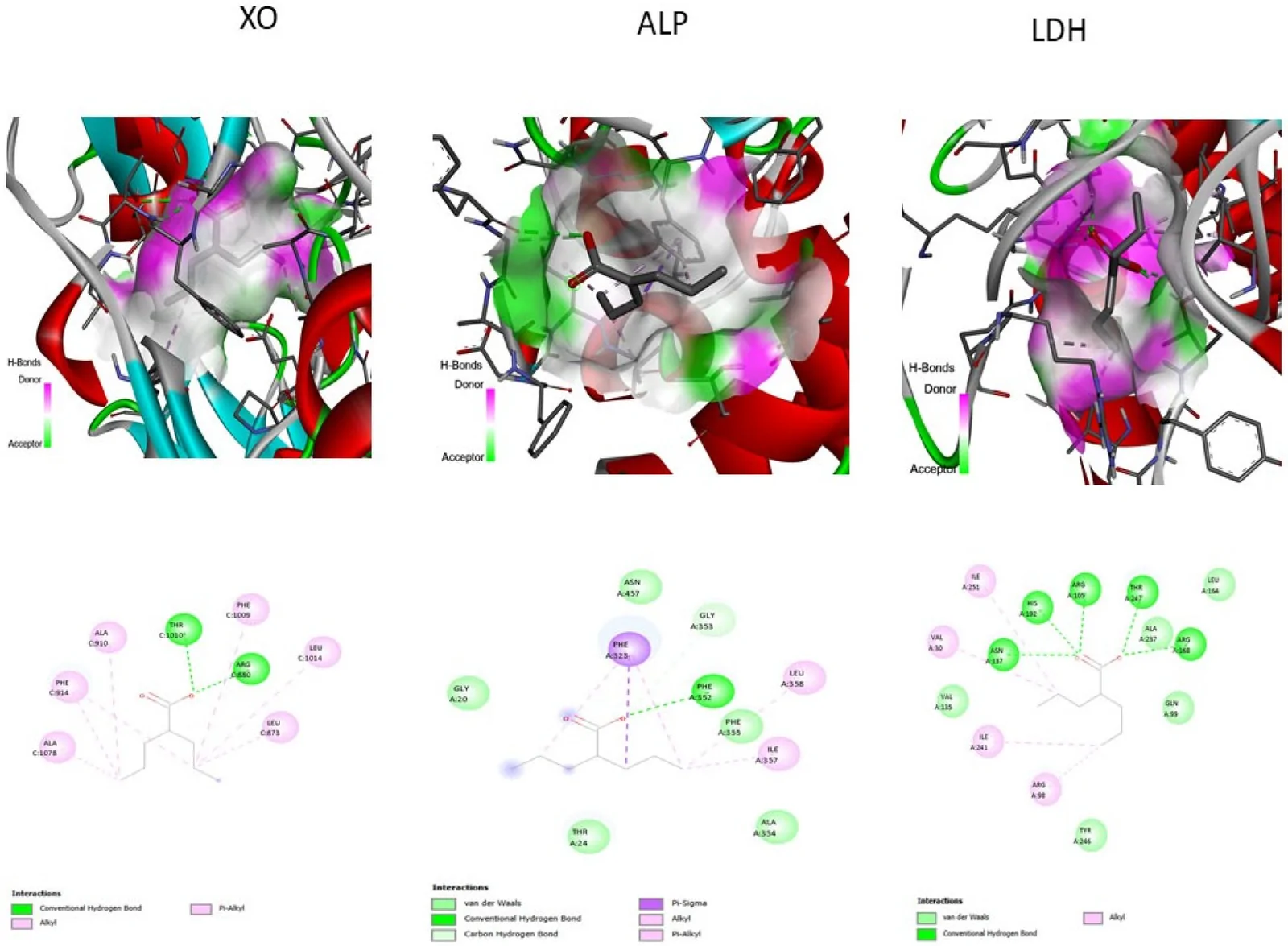
The 3D–2D plots of VPA and XO, ALP, and LDH.

**Figure 8:**
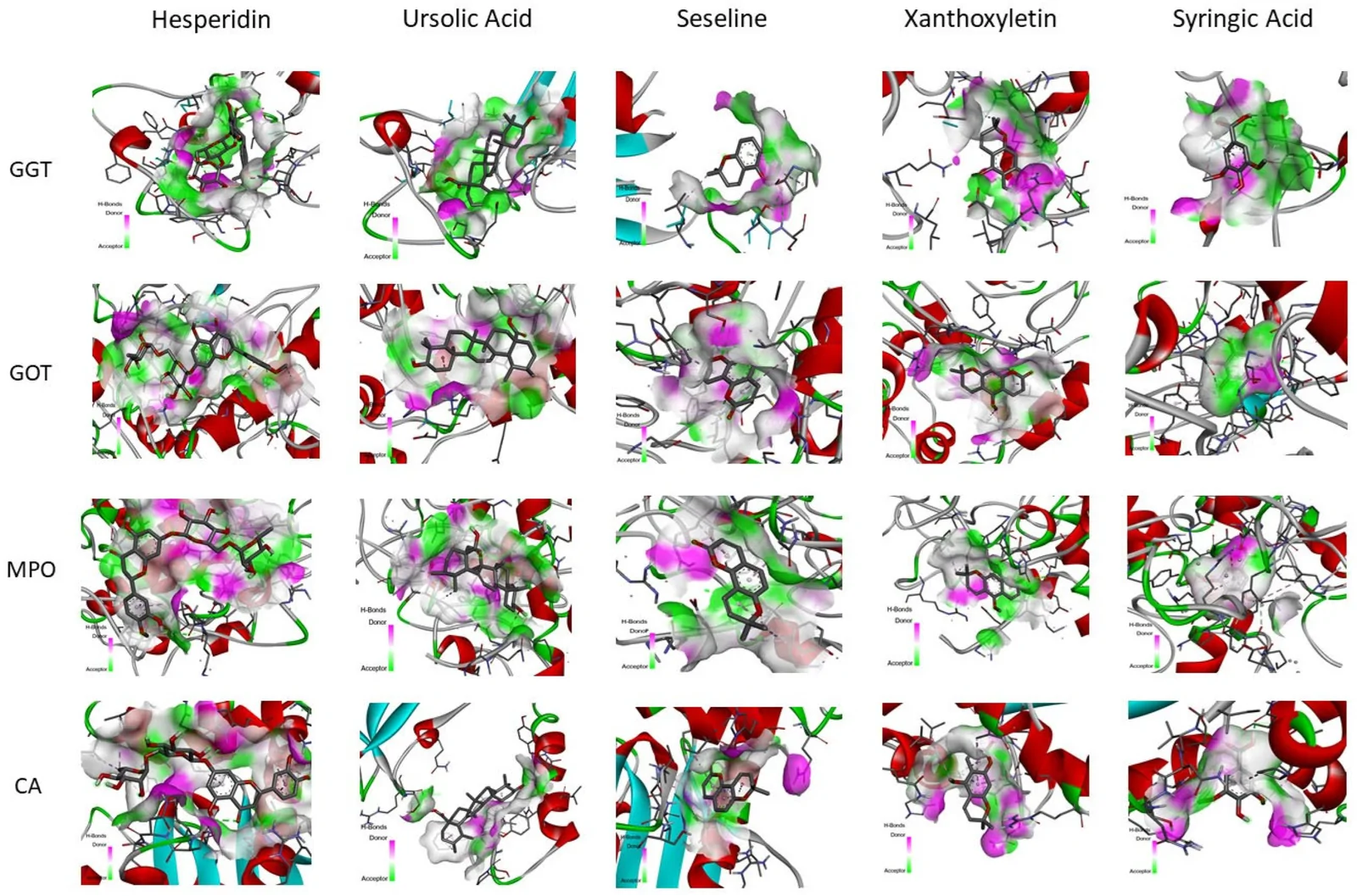
The 3D plots of GGT, GOT, MPO, CA and hesperidin, ursolic acid, seseline, xanthoxyletin and syringic acid.

**Figure 9:**
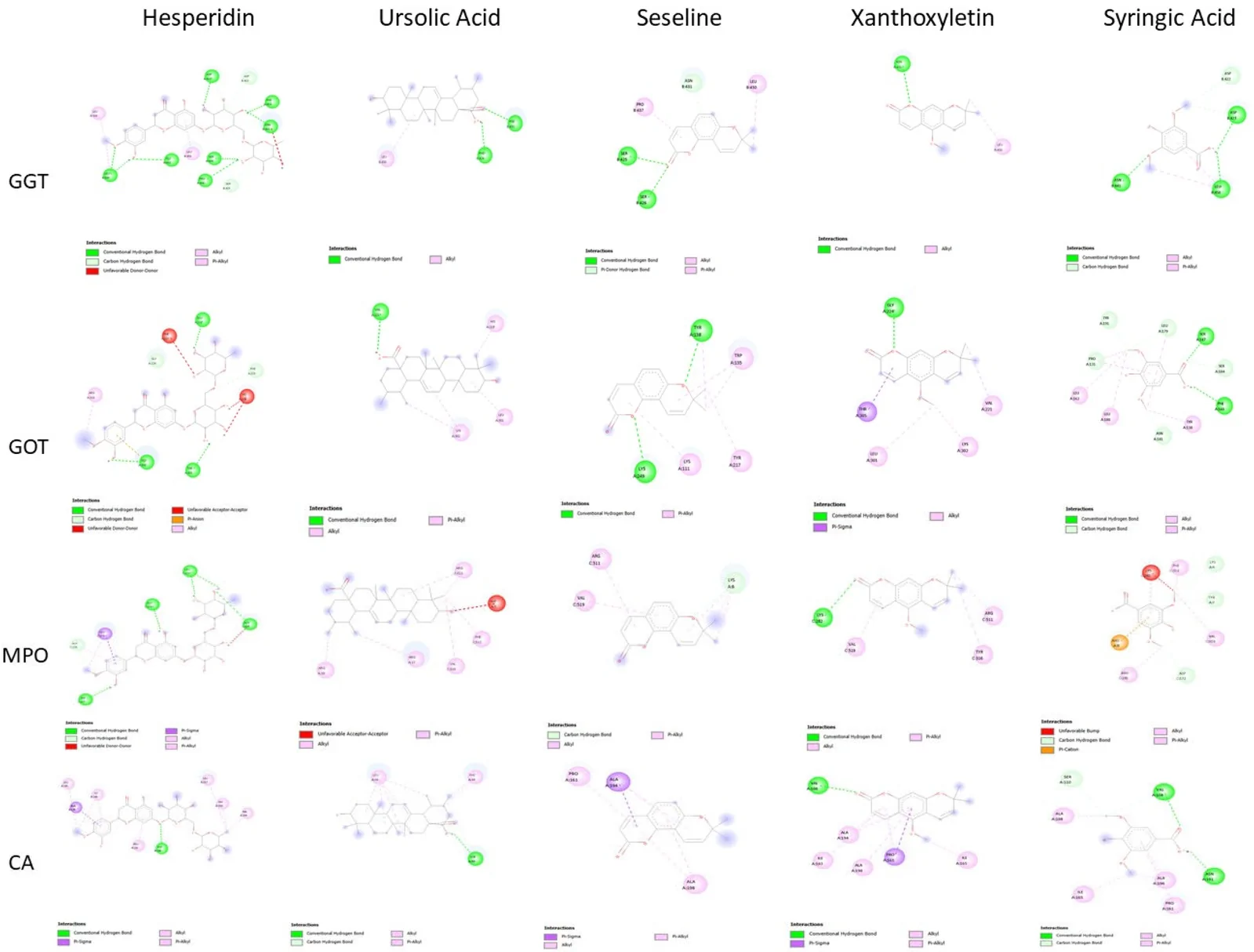
The 2D plots of GGT, GOT, MPO, CA and hesperidin, ursolic acid, seseline, xanthoxyletin and syringic acid.

The 3D–2D plots of VPA and VPA-inhibited enzymes are given in Figure 7. The 3D–2D plots of GGT, GOT, MPO, CA and hesperidin, ursolic acid, seseline, xanthoxyletin and syringic acid were given at Figure 8 and Figure 9. The detailed explanation of all interactions is given in Table 2 and Table 3.

## 3. Discussion

The current study was designed to investigate the harmful effects of SVA on heart tissue, and evaluate the possible protective effects of Moringa extract. Important enzymatic and non-enzymatic parameters, antioxidant defense system and marker enzymes for heart tissue and purine catabolism, as well as membrane stability tests (as glycoprotein levels) were determined.

Oxidative stress is defined as the imbalance between oxidant and antioxidant levels at the cellular level and the impaired redox control at the molecular level [12]. As a results, macromolecules and membrane structure become compromised and distorted. In heart tissue, ROS can be generated by myocytes, endothelial cells and neutrophils. When the mitochondrial level in heart tissue is considered in relation to its function in the body, it’s inevitable that ROS formation and its unwanted effects on the respiratory chain and macromolecules cannot be stopped. The mitochondria are primarily affected by ROS since they lack histone proteins in its chromatin structure. Also, the limited repair capacity of mitochondrial DNA against these hazardous conditions and inadequate exit of superoxide radical out of the mitochondrial membrane makes it a vulnerable target [13,14,15]. These limitations and vulnerabilities can be scavenged via enzymatic/non-enzymatic antioxidant systems.

Cellular redox homeostasis is an important necessary buffering mechanism which prevents excess redox reactions. GSH and its oxidized form (GSSG) are a perfect buffering system against the destruction of the sensitive thiol groups in targeted proteins. They help via glutathionylation and the formation of cysteine cross-bridges [16]. GSH is also important for maintaining protection via GSH-dependent enzyme activities. GPx and GST are dependent on GSH and GSSG existence in the cell media; CAT and SOD also play a role in balancing the antioxidant protection. GPx is much more sensitive than CAT while reducing H_2_O_2_ due to an additive production of SOD from superoxide radical. In the present study, elevated levels of GSH, TOS, ROS and OSI, as well as increased activities of GPx and GST were observed. A decreased TAC levels accompanied by decreased CAT and SOD activities were also observed. The elevation of GSH levels and related enzyme activities may be associated with antioxidant capacity due to SVA-induced oxidative stress. Because cell redox homeostasis must be protected, GSH levels and GSH-related enzymes must provide the defense system to cell. GST play a unique role by quenching electrophilic substances, thus eliminating the elevation of oxidants. Although GPx is sensitive to the existence of H_2_O_2_, its role for detoxification of lipid hydroperoxides is critical. Therefore, the observed elevation of these parameters and the diminishment of other antioxidant enzymes activities are in parallel to previous studies on experimental animals and drug usage in humans [17,18,19]. The administration of Moringa reversed these effects on SVA group. The compounds like ascorbic acid and sulfur containing compounds may have balanced the GSH levels and related enzymatic systems. In addition to these, Moringa contains flavonoids and other strong antioxidant molecules [7,8] that might have ameliorated antioxidant capacity of cell media and related enzyme activities. Findings on levels of TAC, TOS, ROS and OSI alterations also support the antioxidant power of this plant.

Na^+^/K^+^-ATPase is an enzyme embedded in the sarcolemma membrane. In cardiomyocytes, this enzyme helps balance the electrochemical potential across the inner and outer membranes (as sodium and potassium ions move into the cell). The relationship between this enzyme activity and cardiovascular diseases (such as aorta contraction), has been well documented for decades [20,21]. Oxidative stress related membrane disruption can be accepted as the reason for activity loss [10,22,23]. Glycoprotein levels, such as hexose and hexosamine were determined to establish the possible damage to the membrane structure. Based on the current findings, heart hexose and hexosamine levels were increased and Na^+^/K^+^-ATPase activities were decreased after SVA administration. The administration of Moringa restored the membrane integrity. It can be assumed that the antioxidant molecules found in Moringa may have attenuated oxidative conditions and helped restore membrane stability.

The combination of plasma proteins and chlorinated oxidant substances like hypochlorous acid constitute the AOPPs. These molecules are strongly associated to coroner artery diseases, atherosclerosis and cardiomyocytes death [24,25]. MPO is indirectly associated with AOPP formation. This enzyme catalyzes the reaction between hydrogen peroxide and halogens to form hypochlorous acid and other oxidant substances. Due to high lysine and arginine contents, MPO can react with negative charged proteins like albumin or extracellular matrix components [26]. NO is essential for the cardiovascular system arrangement, such as coronary vasodilation and heart muscle contraction. Nevertheless, oxidants rapidly react with NO to form new oxidant molecules like peroxynitrite, that ultimately render NO toxic in heart tissue [15,27]. According to the present results, MPO activities coupled with AOPP and NO levels were elevated in SVA given group. The ameliorative effect of Moringa on antioxidant capacity may have decreased MPO activity and as well down regulated/halted AOPP and NO-derived oxidant formation in SVA given group.

XO is found in the purine catabolism pathway, and is important for uric acid formation. Besides its basic function, uric acid is accepted as protector and antioxidant against cardiovascular and neurological diseases, hydrogen peroxide scavenger and metal ion chelator [26]. Although ALP is mainly associated with bone and heart, recent studies revealed that ALP is an inflammation marker like C-reactive protein in cardiovascular diseases [28]. LDH is an important enzyme found in the glycolytic pathway; it converts pyruvate to lactate. In the present study, decreased activities of XO, ALP and LDH in SVA given group were observed and revealed how SVA altered their structure as seen in Figure 6, Figure 7 and Figure 8. Moreover, diminished XO activity may be related to increased antioxidant need after SVA administration. The increased NO level of the SVA given group can support decreased ALP activity to prevent possible elevation of inflammation. Moreover, LDH is found as LDH-A isozyme in heart tissue. Lactate has a role as signaling molecule for the differentiation of vascular smooth muscle cells. Hence, decreased LDH activity at these conditions may be related to this approach [29] as well as indirect interaction of SVA with the enzyme. The present findings are in agreement with these approaches. In addition, Magaji et al. [30] proved that SVA have caused inflammation and increased cytokine levels at SVA given lung tissue. It is worthwhile to suggest that the antioxidant property and anti-inflammatory effect of Moringa prevented deleterious biochemical alterations in the SVA + Moringa group compared to the SVA group.

CA regulates the pH balance, and CO_2_ formation or excretion. The elevation of CA activity has been related to increased ion channel activities. Due to this reason, CA inhibitors have been used for prevention of heart diseases [31,32]. GGT is responsible for protecting the GSH concentration and accepting amino acid into cell media. During this metabolic event, GSH is decomposed and Cys, Cys-Gly parts are formed. Cys-Gly can directly react with ferrous ions to form ferric ions; leading to the oxidation of LDL and plaque formation that result in atherosclerosis [33]. GOT is one of the important transaminases and helps in the transfer of NADH to mitochondria through the malate-aspartate shunt by supplying aspartate. Owing to this, GOT is an important marker for heart disease [34]. These three enzymes (CA, GGT and GOT) were found to be increased after SVA administration. However, treatment with Moringa extracted resulted to a restored activity of the enzymes. These may be related to the powerful compounds present in Moringa and the inhibition of these enzymes as shown in Table 3, and Figure 7 and Figure 8.

PCA analysis revealed that the first two principal components possessed 84.39% of the total variance, indicating that the measured biochemical parameters adequately represented the overall physiological and oxidative status of the experimental groups. The strong clustering of oxidative stress-related biomarkers, including ROS, TOS, OSI, AOPP, MPO and NO, suggests that these variables are biologically interconnected and respond in a coordinated manner to the experimental conditions. In contrast, antioxidant and protective parameters such as SOD, CAT, TAC, Na^+^/K^+^-ATPase and LDH activity were positioned in the opposite direction on the PCA loading plot, indicating an inverse relationship with oxidative damage markers. This pattern supports the hypothesis that activation of oxidative pathways is accompanied by suppression or exhaustion of antioxidant defense mechanisms. Furthermore, the strong positive association between glycoprotein levels and oxidative stress indicators suggests that alterations in glycoprotein levels may be linked to oxidative injury and cellular stress responses. Overall, PCA demonstrated a distinct clustering pattern associated with Moringa treatment. It highlighted the biochemical parameters strongly contributing to the observed multivariate variation.

Based on these results, the treatment of SVA-induced heart tissue injury with Moringa oleifera extract is associated with favorable changes in selected biochemical parameters that are related to oxidative stress conditions and purine metabolism.

### Limitations

The main limitation of this study is that bioassay-guided fractionation, isolation of individual constituents, and structural elucidation would be necessary to identify and confirm the specific compound(s) responsible for the observed activity. However, these experiments are beyond the scope of the present study, which was primarily designed to investigate the protective effect of *Moringa oleifera* leaf extract against valproic acid-induced heart damage, and as well explore the associated biochemical and in silico findings. The findings suggest a potential protective role of *Moringa oleifera* extract, although direct cardiac injury and histopathological endpoints were not assessed. Additionally, the identification of the specific bioactive constituents is an important limitation of the present study and will be a direction for future research. Also, the absence of formal randomization is a limitation in this study.

## 4. Materials and Methods

### 4.1. Preparation of Plant Extract

Fresh leaves of *M. oleifera* were collected from farms in Sokoto town, Sokoto Province/Nigeria, around July 2018. This plant was described by Taxonomist Umar Abdullahi (PhD) (herbarium number: UDUS/VS/2011/31) from the Botany Unit, Department of Biological Sciences, Usmanu Danfodiyo University, Sokoto.

The leaves were prepared to make an ethanolic extract. The powder of dried fresh leaves (20 g) was extracted in a Soxhlet apparatus using 70% ethyl alcohol (300 mL). The extract was boiled until the color turned to light green. The extract was filtered and then evaporated using a rotary evaporator (Buchi, B-491, BUCHI Labortechnik AG, BUCHI Labortechnik AG). The obtained residues were kept at −20 °C until the experiment was performed. Extraction with ethanol produced 36.43 g of dry extract from 100 g of powdered leaves, corresponding to an extraction yield of 36.43% (*w*/*w*). GC analysis was also performed for the plant extract.

### 4.2. Ethical and Experimental Procedure

The ethical approval was obtained from the Experimental Animal Local Ethical Committee of Marmara University (Approved Number: 28.2024mar). Female Sprague-Dawley rats (forty-six rats and 3.0–3.5 months old, 280–300 g) were preferred for this study. They had access to standard pellets and water ad libitum. The animals were split into four groups as follows: control animals received physiological saline (*n* = 8), Moringa-given group (*n* = 15), SVA administered group (*n* = 8) and SVA + Moringa-given group (*n* = 15) to which SVA and Moringa extract were administered at the same dose and application time. SVA and Moringa extract were dissolved at physiological saline (0.9% NaCl) and given to animals by gavage methods. According to the weights of animals, 1 mL of the SVA and Moringa were given at a calculated concentration of 500 mg/kg and 300 mg/kg respectively. The control group received 1 mL physiological saline at the same time of treatment with other groups. The treatments (administration of 0.9% NaCl, Moringa extract and SVA) were administered for 15 days. After one night fasting, all animals were sacrificed under ketamine anesthesia (80 mg/kg, with 10 mg/kg xylazine hydrochloride, i.m.) and heart tissues were taken.

### 4.3. Biochemical Experiments

The heart tissues were weighed and then homogenized to make up 10% [*w*/*v*] homogenates by using cold physiological saline. The homogenates were centrifuged at 10,000 rpm for 10 min at +4 °C. The supernatants were collected and stored at −80 °C for before analysis. Biochemical parameters like GSH [35], AOPP [25], TAC and TOS [36,37], ROS [38] and OSI, calculated as the % [TOS]/[TAC] concentrations], CAT [39], SOD [40], GPx [41], GST [42], GGT [43], GOT [44], MPO [45], XO [46], NO [47], LDH [48], Na^+^/K^+^-ATPase [49], CA [50], hexose and hexosamine [51], ALP [52] were determined in heart tissue homogenate. The protein levels of all samples were determined according to the method of Lowry et al. [53]. The GSH, TAC, TOS, NO, hexose and hexosamine levels were determined spectrophotometrically. Similarly, CAT, GPx, GST, XO, LDH activities and AOPP were measured spectrophotometrically according to standard methods. Estimation of CAT and GPx activities were based on the alteration of hydrogen peroxide and NADPH concentrations, respectively. The rest of the enzymes were determined as colored-final product absorbance. Hexose and hexosamine levels were determined after protein-free supernatants were prepared. This was achieved via a process of protein hydrolysis by reacting the obtained supernatants of homogenates in acidic medium at 100 °C for 3 h.

### 4.4. In Silico Studies

The molecular docking studies were performed on different enzymes and VPA group. This was aimed to investigate how VPA interacted with the structure of these enzymes. The important natural compounds found in Moringa chosen were hesperidin (PubChem ID: 10621), seselin (PubChem ID: 68229), syringic acid (PubChem ID: 10742), ursolic acid (PubChem ID: 64945) and xanthoxyletin (PubChem ID: 66548). They were downloaded as .sdf format (https://pubchem.ncbi.nlm.nih.gov/). The XO (PDB ID: 1FIQ), ALP (PDB ID: 7PQ8), LDH (PDB ID: 4AJP), GGT (PDB ID: 4GG2), GOT (PDB ID: 1AJS), MPO (PDB ID: 1DNU) and CA (PDB ID: 9S4G) were chosen and downloaded as .pdb format from the Protein Data Bank website (https://www.rcsb.org). The VPA molecule was downloaded as .pdb format from the GoDrugBank website [https://go.drugbank.com/]. The energy minimization of these compounds was undergone via the Universal Field Force (UFF) method with Avogadro program. AutoDock Vina (Version 4.2) and PyMOL (3.1.6.1) Software (free version) were used to determine the interactions. The visualization of these structures was designed via Discovery Studio 2025 Client Software.

The RMSD values were calculated to validate the molecular docking protocol for each enzyme. The RMSD values are considered indicative of the method’s confidence if they are under 2 Å. The calculations were performed by determining the conformation between the ligand in the original crystal and the best-scoring pose.

### 4.5. Statistical Analyses

All the biochemical parameters, PCA loadings, eigenvalues results, and Pearson r correlation matrix were evaluated and prepared using the GraphPad Prism 9.5 program. The results were assessed with an unpaired *t*-test and analysis of variance (ANOVA) followed by Tukey’s multiple comparison tests. A value *p* < 0.05 was accepted as statistically significant. The values were expressed as the mean ± standard deviation (SD). Moreover, the technical replicates (which is 3 per animal here) were used for improving measurement reliability but did not affect any of the statistical sample size.

## 5. Conclusions

Due to the importance of the heart in the body, the interaction of drugs with this organ must be carefully evaluated. This study investigated the effect of SVA-induced heart injury through oxidative stress, changes in antioxidant defense, lipid peroxidation, and related biochemical parameters. According to the outcome, SVA was observed to directly affect important enzyme activities, oxidant/antioxidant balance, signaling molecules, muscle parameters, etc. Moringa, as a powerful antioxidant food/plant, reversed these negative effects in heart tissue. Thus, Moringa can strongly prevent oxidative stress/conditions induced by SVA, and as well restore antioxidant parameters in heart tissue.

## Figures and Tables

**Table 1:** The information for molecular docking studies (related to Figure 6).

Enzyme	PDB ID	RMSD Value	Docking Software	Crystallogpraphic Ligand
XO	1FIQ	0.76	AutoDock Vina-PyMol	The inhibitor ligand as indicated in the PDB website
ALP	7QP8	1.25	AutoDock Vina-PyMol	The inhibitor ligand as indicated in the PDB website
LDH	4AJP	1.57	AutoDock Vina-PyMol	The inhibitor ligand as indicated in the PDB website
GGT	4GG2	0.73	AutoDock Vina-PyMol	The inhibitor ligand as indicated in the PDB website
GOT	1AJS	1.74	AutoDock Vina-PyMol	The inhibitor ligand as indicated in the PDB website
MPO	1DNU	1.78	AutoDock Vina-PyMol	The inhibitor ligand as indicated in the PDB website
CA	9S4G	1.33	AutoDock Vina-PyMol	The inhibitor ligand as indicated in the PDB website

**Table 2:** The molecular docking results of valproic acid and inhibited enzymes. BE: binding energy, IAA: interacted amino acids, IT: interaction type.

Substance	Enzyme								
	XO			ALP			LDH		
	BE (kcal/mol)	IAA	IT	BE (kcal/mol)	IAA	IT	BE (kcal/mol)	IAA	IT
Valproic Acid	−5.0	Leu873 Arg880 Ala910 Phe914 Phe1009 Thr1010 Leu1014 Ala1078	Conventional hydrogen bond, alkyl and π-alkyl	−4.4	Phe323 Phe352 Gly353 Ile357 Leu358	Conventional hydrogen bond, carbon hydrogen bond, van der Waals, π -alkyl, π- sigma, alkyl	−5.0	Val30 Arg98 Arg105 Asn138 Arg168 His192 Ile241 Thr247	Conventional hydrogen bond, van der Waals, alkyl

**Table 3:** The molecular docking results of some natural compounds of Moringa and the inhibited enzymes. BE: binding energy, IAA: interacted amino acids, IT: interaction type.

Natural Product in Moringa and		Enzyme											
PubChem IDs		GGT			GOT			MPO			CA		
		BE (kcal/mol)	IAA	IT	BE (kcal/mol)	IAA	IT	BE (kcal/mol)	IAA	IT	BE (kcal/mol)	IAA	IT
Hesperidin	10621	−9.4	Thr381 Asn401 Leu402 Tyr403 Asp422 Asp423 Phe424 Val435 Ser438 Asn441 Ser452 Gly474 Leu509	Conventional and carbon hydrogen bonds, alkyl and π -alkyl, π-sigma, amide- π stacked, π-anion, unfavorable donor-donor	−9.2	Ala39 Val41 Val49 Trp48 Val53 Glu57 Trp140 Asn264	Conventional hydrogen bonds, van der Waals, alkyl and π -alkyl, unfavorable acceptor-acceptor	−9.0	Leu223 Ser227 Ala228 Leu373 Glu374 Gln409	Conventional and carbon hydrogen bonds, unfavorable donor-donor, alkyl, Pi-sigma, Pi-alkyl	−8.0	Val154 Leu157 Trp158 Pro161 His164 Ile165 Leu190 Ala194	Conventional hydrogen bond, alkyl, Pi-alkyl, Pi-sigma
Ursolic acid	64945	−7.8	Leu402 Asn431 Asn441 Leu450	Conventional hydrogen bond, van der Waals, alkyl, unfavorable donor-donor	−8.8	Leu16 Val17 Trp140 Leu382 Pro383	van der Waals, alkyl and π -alkyl	−7.4	Asp5 Arg17 Arg18 Arg511 Phe512 Val519	Unfavorable acceptor-acceptor, alkyl and Pi-alkyl	−8.0	Phe34 Leu36 Ser84	Conventional and carbon hydrogen bonds, alkyl and Pi-alkyl
Seselin	68229	−7.4	Asp423 Met453	Conventional hydrogen bond, van der Waals, π-anion	−8.1	Val17 Phe18 Val37 Trp140 Ala224 Lys258	Conventional hydrogen bonds, van der Waals, alkyl and π -alkyl, π- π stacked, π-cation	−6.9	Lys6 Arg511 Val519	Carbon hydrogen bond, alkyl and Pi-alkyl	−6.8	Pro161 Ala194 Ala198	Alkyl, Pi-alkyl, Pi, sigma
Xanthoxyletin	66548	−6.9	Leu402 Asp423 Ser452 Met453	Conventional hydrogen bond, van der Waals, π-anion, alkyl	−7.5	Phe18 Trp140 Ala224 Lys258 Arg266	Conventional hydrogen bonds, van der Waals, π -alkyl, π- π stacked	−6.5	Lys282 Tyr316 Arg511 Val519	Carbon hydrogen bond, alkyl and Pi-alkyl	−6.3	Ile107 Val108 Pro161 Ile165 Ala194 Ala198	Conventional hydrogen bond, alkyl, Pi-alkyl, Pi-sigma
Syringic acid	10742	−5.7	Leu384 Ala546 Ala557 Ala567 Pro566	Conventional and carbon hydrogen bonds, van der Waals, alkyl and π -alkyl, unfavorable donor-donor	−6.1	Thr109 Trp140 Asn194 Ala224 Tyr225 Lys258	Conventional hydrogen bonds, van der Waals, alkyl and π -alkyl, π- π stacked, π-cation	−5.0	Lys6 Tyr7 Arg8 Asp172 Arg281 Lys282 Phe512 Val519	Carbon hydrogen bond, alkyl and Pi-alkyl, Pi-cation, unfavorable bump	−5.1	Val108 Ser110 Pro161 Ile165 Asn191 Ala194 Ala198	Conventional and carbon hydrogen bonds, alkyl and Pi-alkyl

## Data Availability

The raw data supporting the conclusions of this article will be made available by the authors on request.

## Abbreviations

The following abbreviations are used in this manuscript:

ALP	Alkaline phosphatase
AOPP	Advanced oxidized protein products
BE	Binding energy
CA	Carbonic anhydrase
CAT	Catalase
VPA	Valproic acid
GGT	Gamma glutamyl transferase
GOT	Glutamate oxalacetate transminase
GPx	Glutathione peroxidase
GSH	Reduced glutathione
GST	Glutathione-S-transferase
IAA	Interacted amino acids
IT	Interaction type
LDH	Lactate dehyrogenase
MPO	Myeloperoxidase
Na^+^/K^+^-ATPase	Sodium potassium ATPase
NO	Nitric oxide
OSI	Oxidative stress index
PCA	Principal component analysis
ROS	Reactive oxygen species
SOD	Superoxide dismutase
SVA	Sodium valproate
TAC	Total antioxidant capacity
TOS	Total oxidant status
XO	Xanthine oxidase

## References

[1] Magaji U.F., Miftari S., Saçan Ö., Yanardağ R. (2023). Effect of antiepileptic drug sodium valproate on stomach tissue glycoproteins: The protective role of moringa extract. Arch. Epilepsy.

[2] Tarahomi P., Arab M., Seyedinia S.A., Rahmani M., Rashidy-Pour A., Vafaei A.A., Raise-Abdullahi P. (2025). Vitamin C and gallic acid ameliorate motor dysfunction, cognitive deficits, and brain oxidative stress in a valproic acid-induced model of autism. Brain Behav..

[3] Terim Kapakin K.A., Bolat İ., İskender H., Dokumacioğlu E., Manavoğlu Kirman E., Bolat M., Gül M. (2025). Protective effects of curcumin against valproic acid induced brain kidney and liver damage in rats. Drug Chem. Toxicol..

[4] Chaudhary S., Parvez S. (2012). An in vitro approach to assess the neurotoxicity of valproic acid-induced oxidative stress in cerebellum and cerebral cortex of young rats. Neuroscience.

[5] Liu Y., Li S., Zhang Z., Lv Z., Jiang H., Tan X., Tan X., Liu F. (2017). Effects of valproic acid on sympathetic activity and left ventricularmyocardial remodelling in rats during pressure overload. Turk. J. Med. Sci..

[6] Daniels T., Gallagher M., Tremblay G., Rodgers R.L. (2004). Effects of valproic acid on cardiac metabolism. Can. J. Physiol. Pharmacol..

[7] Aja P.M., Nwachukwu N., Ibiam U.A., Igwenyi I.O., Offor C.E., Orji U.O. (2014). Chemical constituents of Moringa oleifera leaves and seeds from Abakaliki, Nigeria. Am. J. Phytomed. Clin. Ther..

[8] Liu R., Liu J., Huang Q., Liu S., Jiang Y. (2022). *Moringa oleifera*: A systematic review of its botany, traditional uses, phytochemistry, pharmacology and toxicity. J. Pharm. Pharmacol..

[9] Dağsuyu E., Magaji U.F., Saçan Ö., Yanardağ R. (2023). Moringa oleifera ethanolic extract prevents oxidative damage on lens caused by sodium valproate used in epilepsy treatment. Eur. J. Biol..

[10] Ertik O., Magaji U.F., Sacan O., Yanardag R. (2023). Effect of *Moringa oleifera* leaf extract on valproate-induced oxidative damage in muscle. Drug Chem. Toxicol..

[11] Magaji U.F., Sacan O., Yanardag R. (2022). Antilipase, antiacetylcholinesterase and antioxidant activities of Moringa oleifera extracts. Rom. Biotechnol. Lett..

[12] Sies H. (2020). Oxidative stress: Concept and some practical aspects. Antioxidants.

[13] Kim S.H., Kim H. (2018). Inhibitory effect of astaxanthin on oxidative stress-induced mitochondrial dysfunction-A mini-review. Nutrients.

[14] Sawyer D.B., Colucci W.S. (2000). Mitochondrial oxidative stress in heart failure. Circ. Res..

[15] Tsutsui H., Kinugawa S., Matsushima S. (2011). Oxidative stress and heart failure. Am. J. Physiol.—Heart Circ. Physiol..

[16] Csiszár J., Horváth E., Bela K., Gallé Á. (2016). Glutathione-related enzyme system: Glutathione reductase [GR], glutathione transferases [GSTs] and glutathione peroxidases [GPXs]. Redox State as a Central Regulator of Plant-Cell Stress Responses.

[17] Tunali S., Kahraman S., Yanardag R. (2015). Vitamin U, a novel free radical scavenger, prevents lens injury in rats administered with valproic acid. Hum. Exp. Toxicol..

[18] Emekli-Alturfan E., Alev B., Tunali S., Oktay S., Tunali-Akbay T., Ozturk L.K., Yanardag R., Yarat A. (2015). Effects of edaravone on cardiac damage in valproic acid induced toxicity. Ann. Clin. Lab. Sci..

[19] Zhang Y.J., Zhang M., Wang X.C., Yu Y.H., Jin P.J., Wang Y. (2011). Effects of sodium valproate on neutrophils’ oxidative metabolism and oxidant status in children with idiopathic epilepsy. Zhonghua Er Ke Za Zhi Chin. J. Pediatr..

[20] Ellingsen Ø., Holthe M.R., Svindland A., Aksnes G., Sejersted O.M., Ilebekk A. (1994). Na,K-pump concentration in hypertrophied human hearts. Eur. Heart J..

[21] Yazaki Y., Fujii J. (1972). Depressed Na-K-ATPase activity in the failing rabbit heart. Jpn. Heart J..

[22] Ertik O., Saçan Ö., Kabasakal L., Şener G., Yanardağ R. (2021). Protective effect of chard extract on glycoprotein compounds and advanced oxidation protein product levels in diabetic rat livers. Experimed.

[23] Sacan O., Turkyilmaz I.B., Bayrak B.B., Mutlu O., Akev N., Yanardag R. (2016). Zinc supplementation ameliorates glycoprotein components and oxidative stress changes in the lung of streptozotocin diabetic rats. BioMetals.

[24] Valente A.J., Yoshida T., Clark R.A., Delafontaine P., Siebenlist U., Chandrasekar B. (2013). Advanced oxidation protein products induce cardiomyocyte death via Nox2/Rac1/superoxide-dependent TRAF3IP2/JNK signaling. Free Radic. Biol. Med..

[25] Witko-Sarsat V., Friedlander M., Capeillère-Blandin C., Nguyen-Khoa T., Nguyen A.T., Zingraff J., Jungers P., Descamps-Latscha B. (1996). Advanced oxidation protein products as a novel marker of oxidative stress in uremia. Kidney Int..

[26] Yu W., Cheng J.D. (2020). Uric acid and cardiovascular disease: An update from molecular mechanism to clinical perspective. Front. Pharmacol..

[27] Takimoto E., Kass D.A. (2007). Role of oxidative stress in cardiac hypertrophy and remodeling. Hypertension.

[28] Kunutsor S.K., Bakker S.J.L., Kootstra-Ros J.E., Gansevoort R.T., Gregson J., Dullaart R.P.F. (2015). Serum alkaline phosphatase and risk of incident cardiovascular disease: Interrelationship with high sensitivity C-reactive protein. PLoS ONE.

[29] Yang L., Gao L., Nickel T., Yang J., Zhou J., Gilbertsen A., Geng Z., Johnson C., Young B., Henke C. (2017). Lactate promotes synthetic phenotype in vascular smooth muscle cells. Circ. Res..

[30] Magaji U.F., Koroglu P., Coremen M., Bulan O.K., Sacan O., Yanardag R. (2025). Effects of Moringa oleifera extract on biochemical and histological parameters of sodium valproate induced lungs damage. J. Mol. Histol..

[31] Swenson E.R., Kumar A., Kumar N., Alvarez B.V. (2021). Targeting carbonic anhydrases in cardiovascular and pulmonary disease. The Carbonic Anhydrases: Current and Emerging Therapeutic Targets.

[32] Vargas L.A., Pinilla O.A., Díaz R.G., Sepúlveda D.E., Swenson E.R., Pérez N.G., Álvarez B.V. (2016). Carbonic anhydrase inhibitors reduce cardiac dysfunction after sustained coronary artery ligation in rats. Cardiovasc. Pathol..

[33] Ndrepepa G., Kastrati A. (2016). Gamma-glutamyl transferase and cardiovascular disease. Ann. Transl. Med..

[34] Ndrepepa G. (2021). Aspartate aminotransferase and cardiovascular disease—A narrative review. J. Lab. Precis. Med..

[35] Beutler E. (1975). Glutathione in red cell metabolism. A Manual of Biochemical Methods.

[36] Erel O. (2004). A novel automated direct measurement method for total antioxidant capacity using a new generation, more stable ABTS radical cation. Clin. Biochem..

[37] Erel O. (2005). A new automated colorimetric method for measuring total oxidant status. Clin. Biochem..

[38] Zhang Y., Chen J., Ji H., Xiao Z.G., Shen P., Xu L.H. (2018). Protective effects of Danshen injection against erectile dysfunction via suppression of endoplasmic reticulum stress activation in a streptozotocin-induced diabetic rat model. BMC Complement. Altern. Med..

[39] Aebi H. (1984). Catalase in vitro. Methods Enzymol..

[40] Mylroie A.A., Collins H., Umbles C., Kyle J. (1986). Erythrocyte superoxide dismutase activity and other parameters of copper status in rats ingesting lead acetate. Toxicol. Appl. Pharmacol..

[41] Wendel A. (1981). Glutathione peroxidase. Methods Enzymol..

[42] Habig W.H., Jakoby W.B. (1981). Assays for differentiation of glutathione S-transferases. Methods Enzymol..

[43] Szasz G. (1969). A kinetic photometric method for serum gamma-glutamyl transpeptidase. Clin. Chem..

[44] Reitman S., Frankel S. (1957). A colorimetric method for the determination of serum glutamic oxalacetic and glutamic pyruvic transaminases. Am. J. Clin. Pathol..

[45] Wei H., Frenkel K. (1991). In vivo formation of oxidized DNA bases in tumor promoter-treated mouse skin. Cancer Res..

[46] Corte E.D., Stirpe F. (1968). Regulation of xanthine oxidase in rat liver: Modifications of the enzyme activity of rat liver supernatant on storage at 20 degrees. Biochem. J..

[47] Miranda K.M., Espey M.G., Wink D.A. (2001). A rapid, simple spectrophotometric method for simultaneous detection of nitrate and nitrite. Nitric Oxide.

[48] Bais R., Philcox M. (1994). Approved recommendation on IFCC methods for the measurement of catalytic concentration of enzymes. Part 8. IFCC Method for Lactate Dehydrogenase [l-Lactate: NAD+Oxidoreductase, EC 1.1.1.27]. Eur. J. Clin. Chem. Clin. Biochem..

[49] Ridderstap A.S., Bonting S.L. (1969). Na^+^-K^+^-activated ATPase and exocrine pancreatic secretion in vitro. Am. J. Physiol..

[50] Verpoorte J.A., Mehta S., Edsall J.T. (1967). Esterase activities of human carbonic anhydrases B and C. J. Biol. Chem..

[51] Winzler R.J. (1955). Determination of serum glycoproteins. Methods Biochem. Anal..

[52] Walter K., Schütt C. (1974). Acid and alkaline phosphatase in serum. Method. Enzym. Anal..

[53] Lowry O.H., Rosebrough N.J., Farr A.L., Randall R.J. (1951). Protein measurement with the Folin phenol reagent. J. Biol. Chem..

